# Platelet Activation after Presyncope by Lower Body Negative Pressure in Humans

**DOI:** 10.1371/journal.pone.0116174

**Published:** 2014-12-29

**Authors:** Morten Zaar, Chriselda G. Fedyk, Heather F. Pidcoke, Michael R. Scherer, Kathy L. Ryan, Caroline A. Rickards, Carmen Hinojosa-Laborde, Victor A. Convertino, Andrew P. Cap

**Affiliations:** 1 University of Texas Health Science Center at San Antonio, San Antonio, Texas, United States of America; 2 United States Army Institute of Surgical Research, Fort Sam Houston, Texas, United States of America; 3 University of North Texas Health Science Center Fort Worth, Fort Worth, Texas, United States of America; Royal College of Surgeons, Ireland

## Abstract

Central hypovolemia elevates hemostatic activity which is essential for preventing exsanguination after trauma, but platelet activation to central hypovolemia has not been described. We hypothesized that central hypovolemia induced by lower body negative pressure (LBNP) activates platelets. Eight healthy subjects were exposed to progressive central hypovolemia by LBNP until presyncope. At baseline and 5 min after presyncope, hemostatic activity of venous blood was evaluated by flow cytometry, thrombelastography, and plasma markers of coagulation and fibrinolysis. Cell counts were also determined. Flow cytometry revealed that LBNP increased mean fluorescence intensity of PAC-1 by 1959±455 units (*P*<0.001) and percent of fluorescence-positive platelets by 27±18%-points (*P* = 0.013). Thrombelastography demonstrated that coagulation was accelerated (R-time decreased by 0.8±0.4 min (*P* = 0.001)) and that clot lysis increased (LY_60_ by 6.0±5.8%-points (*P* = 0.034)). Plasma coagulation factor VIII and von Willebrand factor ristocetin cofactor activity increased (*P* = 0.011 and *P* = 0.024, respectively), demonstrating increased coagulation activity, while von Willebrand factor antigen was unchanged. Plasma protein C activity and tissue-type plasminogen activator increased (*P* = 0.007 and *P* = 0.017, respectively), and D-dimer increased by 0.03±0.02 mg l^−1^ (*P* = 0.031), demonstrating increased fibrinolytic activity. Plasma prothrombin time and activated partial thromboplastin time were unchanged. Platelet count increased by 15±13% (*P* = 0.014) and red blood cells by 9±4% (*P* = 0.002). In humans, LBNP-induced presyncope activates platelets, as evidenced by increased exposure of active glycoprotein IIb/IIIa, accelerates coagulation. LBNP activates fibrinolysis, similar to hemorrhage, but does not alter coagulation screening tests, such as prothrombin time and activated partial thromboplastin time. LBNP results in increased platelet counts, but also in hemoconcentration.

## Introduction

Central hypovolemia elevates hemostatic activity whether it is provoked by controlled bleeding [1], [2], simulated bleeding by lower body negative pressure (LBNP) [3], [4], or orthostatic stress [5]. The hemostatic system is composed of several components that create a dynamic balance between coagulation and fibrinolysis, to reduce bleeding after injury while maintaining or restoring vascular patency. Both coagulation and fibrinolytic activities are regulated by components released from the vasculature (*e*.*g*., von Willebrand factor (vWF) and tissue-type plasminogen activator (t-PA)), and are likely regulated in part by increased sympathetic activity resulting from central hypovolemia [6], [7]. However, there is also a cell-based component of elevated hemostatic activity in which coagulation is regulated by the surface of activated platelets [8]. There are a number of pathways, including stimulation by catecholamines, which result in platelet activation. Cross-talk between these pathways increases the complexity of analyzing the platelet contribution to coagulation. Activation of platelets results in conversion of glycoprotein IIb/IIIa (GPIIb/IIIa) into its active form, which is central for platelet aggregation [9]. Thus, activated platelets are essential for hemostasis and severe injury usually results in platelet activation [10], but, intriguingly, platelet activation to central hypovolemia independent of massive tissue trauma has not been demonstrated.

In the present study, healthy subjects were exposed to LBNP until presyncopal symptoms developed, and platelet activation was evaluated by flow cytometric detection of the activation-dependent fibrinogen-mimetic antibody, procaspase-activating compound 1 (PAC-1), bound to GPIIb/IIIa, while coagulation and fibrinolytic activities were evaluated by thrombelastography (TEG) and plasma markers. Additionally, cell counts were determined, and circulating catecholamines were measured to evaluate sympathetic activity. We hypothesized that reduced central blood volume in the absence of tissue trauma would stimulate platelet activation.

## Methods

### Subjects

The study was conducted under a protocol reviewed and approved by the U. S. Army Medical Research and Materiel Command Institutional Review Board and in accordance with the approved protocol.

Eight normotensive, nonsmoking, and otherwise healthy subjects (male/female: 4/4, 30±6/28±11 y, 175±11/168±0 cm, and 70±15/59±8 kg) provided written informed consent before study-related activities were performed. Before the study, subjects received both written and verbal information outlining all procedures and risks associated with the experiment, and were familiarized with the laboratory. On the day of the experiment, a physical examination and an evaluation of each subject's medical history were performed by a physician, and a urine pregnancy test was performed to confirm that female subjects were not pregnant. To reduce the risk of atypical cardiovascular responses, subjects were instructed to maintain their sleeping habits and to avoid exercise, alcohol, and the use of autonomic stimulants such as prescription (*e*.*g*., antihistamines and decongestants) or nonprescription (*e*.*g*., caffeine) drugs for 24 h before the study.

### LBNP Procedure

Each subject was instrumented with a 21-gauge needle in an antecubital vein for blood sampling, and with noninvasive devices (electrocardiogram, and finger photoplethysmography (Finometer, Finapres, Amsterdam, The Netherlands)) for continuous cardiovascular monitoring (heart rate, arterial blood pressures, and stroke volume). After instrumentation, the subject was placed supine with the lower body in the LBNP chamber, which was sealed at the level of the iliac crest by a neoprene skirt, and instructed to avoid leg movements.

After 10 min of rest, the LBNP trial began which followed a stepwise intensification of chamber decompression; 5 min at each step of −15, −30, −45, −60, −70, −80, −90, and −100 mmHg. Termination of LBNP was determined by one or more of the following criteria: (1) continuous decline of systolic blood pressure below 80 mmHg, (2), sudden drop in heart rate, or (3) voluntary termination by the subject because of presyncopal symptoms such as dizziness, impaired vision, nausea, sweating, or malaise. LBNP was followed by 10 min of supine recovery, and the subject was only allowed to leave the laboratory when feeling well, ascertained by responses to questions posed by the investigators.

### Blood samples

At baseline and 5 min after LBNP termination, blood was sampled in 3.2% sodium citrate (BD, BD Vacutainer Citrate Tubes), and disodium EDTA (BD, BD Vacutainer K2EDTA Tubes) without and with reduced glutathione (cell counts, and vasoactive hormones and plasma renin activity, respectively). The maximum blood volume removed per subject for this study was 107 ml. Blood samples for plasma analyses were centrifuged (15 min at 2,000 *g* and room temperature (markers of hemostatic activity) or 4°C (vasoactive hormones and renin activity)), where after plasma was immediately isolated and stored at −80°C until analysis (<6 months). Blood samples for analysis of vasoactive hormones and plasma renin activity were placed in wet ice until centrifugation.

### Flow cytometry

Citrated blood (500 µl) was immediately loaded into cryotubes containing 500 µl of 1% paraformaldehyde fixative solution. Paraformaldehyde-fixed blood (10 µl) was incubated with FITC-PAC-1 (15 min at room temperature). Following incubation, samples were further processed using lyse wash assistant (BD FACS Lyse Wash Assistant, BD Bioscience, CA, USA). Data acquisition was performed on BD FACS CANTO using FACSDiva software (BD FACSDIVA 6.0 software, BD FACSCanto Flow Cytometry, BD Biosciences, CA, USA). Process was repeated for isotype control antibody.

### Thrombelastography

Citrated blood (4.5 ml) was analyzed for coagulation competence (TEG Model 5000; Haemoscope Corporation). Within 30 min of collection, 340 µl blood was activated by 10 µl human recombinant tissue factor (1∶500, Innovin, Dade Behring, FL, USA), added to a TEG-cup containing 20 µl 0.2 M CaCl_2_, and analyzed at 37°C for reaction time until initial fibrin formation (R-time), rate of clot formation (α-Angle), maximal amplitude reflecting clot strength (MA), and clot lysis 30 and 60 min after MA (LY_30_ and LY_60_, respectively).

### Plasma markers of hemostatic activity

Citrated plasma was thawed in a 37°C water bath for 15 min and tested for the following coagulation parameters using a Siemens BCS XP automated coagulation analyzer system: prothrombin time (PT; Dade Innovin), activated partial thromboplastin time (aPTT; Dade Actin FS APTT Reagent), vWF ristocetin cofactor activity (vWF:RCo; BC vWF Reagent), coagulation factor VIII (FVIII; Factor VIII Deficient Plasma), Protein C activity (Berichrom Protein C), D-dimer (Innovance D-Dimer Reagent kit). Additional analysis entailed immunoassays to measure vWF antigen (vWF:Ag; vWF ELISA, Imubind American Diagnostica, Inc.), thrombin-antithrombin III complex (TAT; Enzygnost TAT micro kit, Siemens), and t-PA (t-PA ELISA; Imubind, American Diagnostica, Inc.). Analyses for all samples were processed per manufacturer's directions.

### Hematology

Within 2 h of collection, EDTA-stabilized blood was analyzed for cell counts (platelets, red and white blood cells, lymphocytes, monocytes, and basophils), hemoglobin, and hematocrit (CELL-DYN 3700, Abbott Diagnostics).

### Circulating vasoactive hormones and plasma renin activity

Plasma was obtained from EDTA-stabilized blood (7 ml) collected in tubes with reduced glutathione to minimize catecholamine oxidative degradation. Epinephrine and norepinephrine concentrations were measured by ELISA (BA E-5400 2-CAT (epinephrine/norepinephrine) Research ELISA, Rocky Mountain Diagnostics) on a Thermo Labsystems plate reader (iEMS Reader MF). The epinephrine and norepinephrine standard curves were fitted with a four-parameter logistic by nonlinear regression analysis (SigmaPlot) for the calculation of plasma epinephrine and norepinephrine concentrations. Plasma arginine vasopressin was measured by radioimmunoassay after extraction using C18 cartridges (Ultrasensitive-ADH/arginine vasopressin (RK-AR1), ALPCO Immunoassays). The arginine vasopressin standard curve was fitted with a four-parameter logistic by nonlinear regression analysis (SigmaPlot) for the calculation of plasma arginine vasopressin concentration. Plasma renin activity was calculated from the measurement of angiotensin I by radioimmunoassay (GammaCoat Plasma Renin Activity (CA-1553), DiaSorin). The standard curve was fitted with a four-parameter logistic by nonlinear regression analysis (SigmaPlot) for the calculation of plasma angiotensin I concentration in hot (37°C) and cold (4°C) incubated aliquots of plasma. The radioimmunoassay results were used to calculate plasma renin activity (ng of angiotensin I generated per hour of incubation).

### Statistical analysis

Data were evaluated by paired *t*-test or, if the normality test failed, by Wilcoxon Signed Rank Test using SigmaStat software integrated in SigmaPlot (version 11.0; Systat Software). The probability of observing chance effects on the dependent variables of interest are presented as exact *P*-values, and *P*<0.05 was considered to represent statistical significance. Data are presented as mean±SD or, if the normality test failed, as median [range]. To correct for multiple comparisons, data were evaluated for false positives by the Benjamini-Hochberg procedure, using a critical value for a false discovery rate of 0.05.

## Results

At 20±6 min and −64±12 mmHg LBNP, all subjects met at least one of the termination criteria, and displayed increased heart rate (63±7 to 128±19 bpm; *P*<0.001, n = 8) and decreased arterial pressures and stroke volume (systolic: 132±10 to nadir 76±13 mmHg, diastolic: 72±8 to nadir 52±15 mmHg, mean: 96±9 to nadir 60±14 mmHg, and pulse pressure: 60±4 to 24±8 mmHg, and stroke volume: 105±9 to 33±4 ml; all *P*<0.01, n = 8). While blood samples were obtained in all subjects, hematological analysis of all samples was not possible due to various technical limitations. As a result, data are shown for groups of 6–8 subjects in Tables 1–5. The binding of PAC-1 was increased after LBNP as demonstrated by both mean fluorescence intensity (MFI) which increased by 57±15% and percent of fluorescence-positive platelets which increased by 91±62% (Table 1). TEG demonstrated a consistent acceleration of coagulation as R-time decreased by 13±6% and, also, an increase in fibrinolysis (LY_60_; LY_30_ did not change significantly after the Benjamini-Hochberg procedure); these values did not exceed their respective reference ranges for healthy subjects (Table 2). In contrast, LBNP did not affect plasma PT and aPTT, but plasma FVIII and vWF:RCo increased by 17±12% and 13±12%, respectively (Table 3). The mean increase in plasma vWF:Ag did not meet statistical significance; however, plasma vWF:Ag decreased in a single subject with a concomitant decrease in plasma TAT. Plasma Protein C activity, t-PA, and D-dimer increased in all subjects. Platelet count increased in 6 of 7 subjects, whereas other cell counts, although highly variable, increased in all subjects (Table 4). There were no changes in plasma catecholamines or vasopressin, and the increase in plasma renin activity differed greatly between subjects (from 40% to 1370%) (Table 5).

**Table 1:** Platelet activity determined by flow cytometry before and 5 min after lower body negative pressure (post-LBNP) was ceased because of presyncopal symptoms.

	Baseline	Post-LBNP	*P*-value
PAC-1 (MFI)	3558±763	5517±1030*	<0.001
PAC-1 (%)	37±19	64±33*	0.013

Mean fluorescence intensity (MFI) and percent (%) of fluorescence-positive platelets after binding of PAC-1 to activated glycoprotein IIb/IIIa. Mean±SD, n = 6, * significantly different from baseline, using the Benjamini-Hochberg procedure with a false discovery rate of 0.05.

**Table 2:** Whole blood coagulation competence determined by thrombelastography before and 5 min after lower body negative pressure (post-LBNP) was ceased because of presyncopal symptoms.

	Baseline	Post-LBNP	*P*-value
R-time (min)	6.4±1.2	5.5±1.0*	0.001
α-Angle (deg)	65±4	67±6	0.224
MA (mm)	60±6	60±4	0.679
LY_30_ (%)	0.5±0.4	3.9±3.2	0.039
LY_60_ (%)	3.0±1.5	8.9±4.8*	0.034

Reaction time until initial fibrin formation (R-time), rate of clot formation (α-Angle), maximum amplitude reflecting clot strength (MA), and clot lysis after 30 and 60 min (LY_30_ and LY_60_). Mean±SD, n = 8, * significantly different from baseline, using the Benjamini-Hochberg procedure with a false discovery rate of 0.05.

**Table 3:** Plasma markers of hemostatic activity before and 5 min after lower body negative pressure (post-LBNP) was ceased because of presyncopal symptoms.

	Baseline	Post-LBNP	*P*-value
PT (s)	10.7±0.5	10.5±0.3	0.163
aPTT (s)	27.7±2.0	27.1±1.8	0.081
vWF:Ag (IU l^−1^)	1016 [610–1371]	1292 [629–1743]	0.078
vWF:RCo (% d.N.)	115±46	128±47*	0.024
FVIII (IU l^−1^)	830±160	982±253*	0.011
TAT (µg l^−1^)	5.5±1.7	9.2±5.5	0.134
Protein C activity (% d.N.)	199±26	214±26*	0.007
t-PA (ng ml^−1^)	0.1±1.6	1.7±2.3*	0.017
D-dimer (mg l^−1^)	0.25 [0.17–0.51]	0.29 [0.19–0.56]*	0.031
			

Prothrombin time (PT), activated partial thromboplastin time (aPTT), von Willebrand factor antigen (vWF:Ag), von Willebrand factor ristocetin cofactor (vWF:RCo), coagulation factor VIII (FVIII), thrombin-antithrombin III complex (TAT), and tissue-type plasminogen activator (t-PA). Mean±SD and median [range], n = 7 (except for t-PA and D-dimer, n = 6), * significantly different from baseline, using the Benjamini-Hochberg procedure with a false discovery rate of 0.05.

**Table 4:** Hematology before and 5 min after lower body negative pressure (post-LBNP) was ceased because of presyncopal symptoms.

	Baseline	Post-LBNP	*P*-value
Platelets (10^6^ µl^−1^)	239±32	272±19*	0.014
White blood cells (10^6^ µl^−1^)	6.9 [5.2–9.6]	8.8 [6.6–10.3]*	0.016
Lymphocytes (%)	1.9 [1.4–2.7]	2.4 [2.2–3.1]*	0.016
Monocytes (%)	0.56±0.15	0.65±0.17*	<0.001
Basophiles (%)	0.06 [0.03–0.07]	0.07 [0.04–0.11]*	0.016
Hematocrit (%)	36.9±3.0	40.4±4.5*	0.005
Red blood cells (10^9^ µl^−1^)	4.3±0.5	4.7±0.5*	0.002
Hemoglobin (g dl^−1^)	12.7 [10.8–13.9]	13.9 [11.6–15.8]*	0.016

Mean±SD and median [range], n = 7, * significantly different from baseline, using the Benjamini-Hochberg procedure with a false discovery rate of 0.05.

**Table 5:** Circulating vasoactive hormones and plasma renin activity before and 5 min after lower body negative pressure (post-LBNP) was ceased because of presyncopal symptoms.

	Baseline	Post-LBNP	*P*-value
Epinephrine (pg ml^−1^)	61±15	58±23	0.766
Norepinephrine (pg ml^−1^)	288±81	410±194	0.097
Vasopressin (pg ml^−1^)	1.1±0.3	1.8±1.4	0.178
Renin activity (ng ml^−1^ h^−1^)	1.1 [0.6–3.5]	2.7 [1.1–13.2]*	0.008

Mean±SD and median [range], n = 8, * significantly different from baseline, using the Benjamini-Hochberg procedure with a false discovery rate of 0.05.

## Discussion

Consistent with our hypothesis, LBNP-induced presyncope in young and healthy humans resulted in platelet activation as demonstrated by an increased binding of PAC-1 detected with flow cytometry, indicating that platelet aggregation is enhanced. Similarly, coagulation and fibrinolysis were activated as demonstrated by both TEG and plasma markers (vWF, FVIII, protein C activity, and t-PA). The elevated hemostatic activity resulted in a minor increase in plasma degradation products of cross-linked fibrin polymers (D-dimer). While TEG revealed that LBNP accelerates tissue factor-activated coagulation, this change was not evident in plasma PT and aPTT results. Hemoconcentration developed, but its implication for hemostatic competence was not evaluated.

Under homeostatic conditions, a hemostatic balance is maintained between coagulation and anticoagulation to avoid thrombosis. After injury, however, enhancement of coagulation is vital to reducing the risk of exsanguination. The hemostatic system is complex, and initiation of coagulation includes both activation of platelets and release of coagulation components from the vasculature and spleen [7], [8], [11]. Several pathways lead to platelet activation, such as stimulation of α_2_-adrenoreceptors by catecholamines, adhesion of GPIb-V-IX on platelet surface to vWF, GPVI and GPIa to collagen at a site of injury, and stimulation of P2Y_12_ receptors by adenosine diphosphate [9]. During activation, platelets undergo a shape change following activation of GPIIb/IIIa, which creates the foundation for platelet aggregation and formation of a platelet plug [9]. The importance of GPIIb/IIIa activation is demonstrated in patients suffering from Glanzmann's thrombasthenia whose platelets fail to aggregate due to a lack of functional GPIIb/IIIa, causing excessive hemorrhage after trauma or surgery [12]. PAC-1 binds to activated GPIIb/IIIa and, in the present study, the increased PAC-1 binding after LBNP demonstrates that platelets were activated and that their aggregation ability was enhanced. In healthy adults exposed to presyncope by combined head-up tilt and LBNP, platelet aggregation is elevated, as evident by impedance aggregometry [3].

Whole blood coagulation was accelerated after LBNP-induced presyncope as demonstrated by a decrease in tissue factor-activated TEG R-time, supporting reports that coagulation is activated by LBNP [3], [4]. Although a significant decrease in R-time followed presyncope, R-time remained within its reference range, suggesting that even changes within a TEG reference range may have clinical significance and that the diagnostic power of a single-point TEG analysis may be limited. In contrast to TEG, plasma PT and aPTT did not change, which may be because PT and aPTT are determined in platelet-poor plasma, thus platelet-driven changes in coagulation may have been missed. Tissue factor-activated TEG, although typically insensitive to hypercoagulation compared to PT and aPTT, may be capturing the effects of vWF on platelet activation-stimulated cell-driven coagulation, which can be detected by whole blood-based, but not plasma-based, clot assays. Supporting this interpretation, the functionality of vWF increased as reflected by elevated plasma vWF:RCo and, although plasma vWF:Ag did not increase significantly, plasma FVIII increased, demonstrating that the amount of circulating vWF increased, since vWF and FVIII circulate as a complex while inactive [13]. Alternatively, the insensitivity of PT and aPTT to hypercoagulation may be explained by an increase in plasma tissue factor pathway inhibitor (TFPI). Although not measured in the present study, plasma TFPI increased in subjects experiencing presyncope provoked by combined head-up tilt and LBNP [3] and, thus, elevated plasma TFPI may have masked a hypercoagulable effect in plasma-based assays [14]. Whatever the case in our study, tissue factor-activated TEG detects hypercoagulation more sensitively in trauma patients than plasma PT and aPTT [15], suggesting a common mechanism stimulated by central hypovolemia.

Central modulators and markers of coagulation activity changed in response to LBNP. While not reaching statistical significance possibly due to high inter-individual variance, plasma TAT increased. Elevation in coagulation activity was accompanied by similar increases in markers of Protein C activity and t-PA, as did fibrinolysis measured by TEG. Elevated coagulation competence is vital to reduce the risk of exsanguination, but an elevated coagulation competence may be associated with an increased risk of thrombosis. Plasma D-dimer did not increase above 0.5 mg l^−1^, indicating that activation of the hemostatic system by LBNP-provoked presyncope does not increase the risk of venous thromboembolism in young and healthy humans [16], which is consistent with another LBNP study in young and healthy humans [4].

Platelet count increased after LBNP-induced presyncope, which may partly be explained by splenic release of platelets after adrenergic stimulation [11]. The classical opinion is that red blood cells are not released by the human spleen [17], but the spleen may contribute to a minor increase (∼3%), as demonstrated in humans exposed to apnea [18]. A more relevant contribution to the increases in cell counts in the present study is the reduction of plasma volume that occurs as fluid reallocates into the extravascular space of the legs during LBNP [19]. Virchow's triad for thrombogenesis highlights the influence of hemoconcentration on hemostatic activity. Red blood cells may indirectly contribute to venous thromboembolism by increasing blood viscosity and causing platelets to marginate along the vessel wall, and directly by increasing phosphatidylserine expression on platelet cell surfaces which may contribute to thrombin generation [20]. This is supported by stress-induced hypercoagulation resulting from both hemoconcentration and increased hemostatic activity [21]. Considering that hemorrhage results in hemodilution and enhanced coagulation competence [2], further investigation is warranted to evaluate the influence of hemoconcentration on LBNP-induced hypercoagulation.

Epinephrine, norepinephrine, vasopressin, and renin activity are mostly commonly recognized for their vasoconstrictive properties during central hypovolemia, but they also affect coagulation. Epinephrine elevates hemostatic competence by activating platelets [9] and by provoking a release of hemostatic components [7]. In humans, epinephrine infusion causes a release of FVIII from the spleen [11] and FVIII, vWF, and t-PA from endothelial cells and, furthermore, results in increased FVIII and vWF activities [7]. Sympathetic activity increases during LBNP-induced central hypovolemia [22], as demonstrated by a doubling of heart rate in the present study, suggesting that epinephrine may be a central hormone in mediating hypercoagulation after blood loss. However, plasma epinephrine and norepinephrine did not differ from baseline, which may be because these catecholamines are rapidly metabolized (both have a plasma half-life of ∼1 min in normovolemic humans [23]) and were therefore cleared from the circulation when the blood samples were obtained 5 min post-LBNP. Vasopressin and the renin-angiotensin system are important after hemorrhage as they affect blood pressure and volume; however, they may also influence hemostatic competence. Vasopressin elevates the expression of phosphatidylserine on platelet surfaces and enhances thrombin generation [24], and increases fibrinolysis by elevating plasma t-PA [25]. In the present study, however, vasopressin did not differ from baseline. Vasopressin increases considerably in response to severe central hypovolemia (30% reduction of the blood volume) [6], and in subjects who display presyncopal symptoms induced by LBNP, vasopressin increased from 0.5 to 55 pg ml^−1^ [26]. However, vasopressin may be cleared from the circulation rapidly (5 min after LBNP-induced presyncope, vasopressin decreased from 55 to 15 pg ml^−1^ [26]), making it difficult to approximate plasma vasopressin at presyncope in the present study. Release of vasopressin is mediated by left atrial stretch receptors and carotid baroreceptors [27], and in humans, plasma vasopressin relates to the magnitude of decrease in systolic pressure provoked by LBNP, as plasma vasopressin increased to ∼30 pg ml^−1^ in subjects whose systolic blood pressure decreased significantly (from 125 to 50 mmHg), whereas plasma vasopressin remained unchanged in subjects whose systolic pressure decreased minimally (from 125 to 110 mmHg) [28]. In the present study, the nadir systolic pressure was ∼75 mmHg at the time of presyncopal symptoms, which may explain why plasma vasopressin remained unchanged. Plasma renin activity relates to renal sympathetic nerve activity and, similar to vasopressin, plasma renin activity increases during LBNP-induced presyncope in high tolerant subjects (from 1 to 4 ng ml^−1^ h^−1^), but not in low tolerant subjects [29]. Renin converts angiotensinogen to angiotensin I, which is converted to angiotensin II that causes hypofibrinolysis by increasing plasma plasminogen activator inhibitor type 1 and, also, causes platelets to become more sensitive to agonists such as epinephrine and adenosine diphosphate [30]. In the present study, both plasma renin activity and fibrinolysis increased after LBNP-induced presyncope, but as plasma plasminogen activator inhibitor type 1 was not measured, a depressant effect of the renin-angiotensin system on fibrinolysis after presyncope remains to be determined. Net fibrinolytic balance is determined by several factors (*e*.*g*., t-PA, urokinase plasminogen activator, plasminogen activator inhibitor type 1, thrombin-activatable fibrinolysis inhibitor, α_2_-antiplasmin, and α_2_-macroglobulin), but it is uncertain whether all of these factors are readily released into the circulation during central hypovolemia. The present study focused on the responses to acute central hypovolemia and such time-frame may have been insufficient to detect some factors (*e*.*g*., transcriptional regulation of plasminogen activator inhibitor type 1). Regardless, the increased fibrinolytic activity demonstrated by tissue factor-activated TEG in the present study may exacerbate bleeding after trauma and explain the beneficial effect of early treatment with antifibrinolytics, *e*.*g*. tranexamic acid [31].

### Limitations

Difficulties to collect blood as presyncope developed during LBNP resulted in blood samples being collected 5 min after presyncope, which limits the interpretation of the vasoactive hormones and renin activity. Also, the present study was performed in young and healthy subjects, and the hemostatic response to central hypovolemia in combination with disease (*e*.*g*., arthrosclerosis) remains unknown.

### Conclusion

In young and healthy humans, presyncope induced by lower body negative pressure activates platelets, resulting in increased surface exposure of active glycoprotein IIb/IIIa, accelerates coagulation, and elevates fibrinolytic activity. Identification of enhanced coagulation is facilitated by thrombelastography compared to plasma prothrombin time and activated partial thromboplastin time.
